# Safety, tolerance, and pharmacokinetics of salvianolic acid B in healthy Chinese volunteers: A randomized, double-blind, placebo-controlled phase 1 clinical trial

**DOI:** 10.3389/fphar.2023.1146309

**Published:** 2023-04-13

**Authors:** Junlin Cheng, Jun Long, Jingjing Zhang, Le Han, Yunfang Hu, Jianghui Liu, Runze Qiu, Zhibin Zhu, Hongwei Fan

**Affiliations:** ^1^ Department of Clinical Pharmacology Lab, Nanjing First Hospital, Nanjing Medical University, Nanjing, China; ^2^ Nanjing Hongqiao Pharmaceutical Technology Research Institute Co Ltd, Nanjing, China

**Keywords:** salvianolic acid B, safety, tolerance, pharmacokinetics, traditional Chinese medicine injection

## Abstract

**Background:** Salvianolic acid B (Sal B) is one of the main active ingredients of Sa*lvia miltiorrhiza* Bunge. In China, many traditional Chinese medicines have been modified into injections for higher bioavailability and better efficacy. Salvianolic acid injection has been widely used in the clinic.

**Objective:** This phase 1, randomized, double-blind, placebo-controlled, single-center study aimed to evaluate the safety, tolerance, and pharmacokinetics of Sal B injection in healthy Chinese volunteers.

**Methods:** For the single-ascending-dose study, forty-seven healthy volunteers were randomly divided into 25, 75, 150, 200, 250, and 300 mg groups. For the multiple-ascending-dose study, sixteen healthy volunteers were randomly divided into 150 and 300 mg groups. In each group, volunteers were treated with Sal B or placebo randomly. Their safety was evaluated by a skin test, physical examination, vital sign, laboratory examination, 12-lead electrocardiogram, Holter, and clinical symptoms and signs. Blood samples were collected in 75, 150, and 300 mg single-ascending-dose study groups and 150 mg multiple-ascending-dose study groups to determine the concentration of salvianolic acid B.

**Results:** In single-ascending-dose study groups, there were 41 adverse events in 24 cases (51.1%, 24/47). In multiple-ascending-dose study groups, there were 13 adverse events in eight cases (50.0%, 8/16). Sixty-six volunteers received the skin test, and three of them were excluded because of the positive result. Adverse events related to the treatment included increased alanine aminotransferase (4.0%), increased bilirubin (2.0%), increased creatinine kinase-MB (2.0%), increased brain natriuretic peptide (8.0%), increased urine N-acetyl-β-D-glucosidase (4.0%), dizziness (2.0%), and chest discomfort (2.0%). No serious adverse events occurred. No volunteers withdrew from the trial. Peak plasma concentration and the area under the plasma concentration–time curve of salvianolic acid B progressively increased in a dose-dependent manner in 75, 150, and 300 mg single-ascending-dose study groups. There was no accumulation after 5 consecutive days of administration of 150 mg salvianolic acid B.

**Conclusion:** Salvianolic acid B injections administered up to 300 mg in a single dose and 250 mg for 5 consecutive days showed excellent safety and tolerability in healthy Chinese volunteers.

**Clinical Trial Registration:** www.chinadrugtrials.org.cn, identifier CTR20192236

## 1 Introduction

Salvia miltiorrhiza (SM) refers to the dried root and rhizome of *Salvia miltiorrhiza* Bunge. SMis a kind of very popular traditional Chinese medicines (TCMs) that has been extensively applied for thousands of years in East Asia (Su et al., 2015). It has been used to treat cardiovascular diseases (Wu et al., 2020), cerebrovascular diseases (Feng et al., 2019), neurodegenerative diseases (Zhao et al., 2019), diabetes (Jia et al., 2019), etc. Moreover, it was widely used in China due to its safety and efficacy in treating cardiovascular and cerebrovascular diseases (Xu et al., 2018; Feng et al., 2019). The pharmacological properties include anti-inflammatory, anti-oxidant, anti-coagulatory, hypolipidemic, anti-apoptotic, vasodilatory, and angiogenesis-promoting actions (Li et al., 2018b). SM could react directly to the Western cardiovascular drug targets relevant to antithrombotic pathways (i.e., thrombin, coagulation factor Xa, and cyclooxygenase-1) (Zuo et al., 2020). Therefore, it could be exploited as an important complementary medicine preparation for pharmacotherapy of cardiovascular and cerebrovascular diseases.

The active ingredients of SM are mainly water-soluble salvianolic acids, which promote blood flow and improve perfusion (Zhang et al., 2018). Sal B is the most active salvianolic acid with the highest content in water-soluble substances (Bi et al., 2022). Since the 1980s, Chinese scientists have researched the water-soluble components of SM, which was used as a decoction. At least 15 water-soluble chemical components had been isolated named in sequences such as Sal A, B, C, and D (Du et al., 2020). In China, at least three kinds of SM polyphenolic acid injections were used in the clinic, which had effects against oxidative stress, platelet aggregation, coagulation, thrombosis, endothelial dysfunction, and inflammation targeting multiple vascular cell types. Salvianolic B (Sal B) is one of the main active ingredients of SM and scavenges different types of free radicals (Wu et al., 2020). Until now, there is no salvianolic acid B injection on the market.

The limited absorption in the gastrointestinal tract has a detrimental effect on the clinical application of SM; injections can increase the concentration of effective substances *in vivo* and increase the bioavailability (Bi et al., 2016). With the development and widespread use of TCM injections, adverse drug reactions (ADRs) have gradually become a public concern (Guo et al., 2015; Li et al., 2018a; Huang et al., 2021). The extraction progresses from the TCMs and the purity of the injections had been considered as the most important factors for severe ADRs (Ji et al., 2009). The Chinese Pharmacopoeia specifies the content index of Sal B and its preparations. In this study, the research institute developed a new preparation technology to extract Sal B with the purity of 96% on a large scale. We hope to provide a new promising Sal B for the clinical use with higher bioavailability and less ADRs. We completed the evaluation of safety, tolerance, and pharmacokinetics of Sal B injections in healthy volunteers from 2019 to 2021.

## 2 Methods

### 2.1 Drugs and preparations

The dried root and rhizome of *S. miltiorrhiza* Bunge have been identified by Dr. Shengjin Liu (the School of Pharmacy, Nanjing University of Chinese Medicine). A voucher specimen (No. nzy-ds-180423) was deposited at the Chinese Medicine Herbarium of Nanjing University of Chinese Medicine. The extraction of *S. miltiorrhiza* is concentrated and freeze-dried after refining (Patent No: ZL02160771.0). The content of salvianolic acid B in the refined stock solution is higher than 96%. A quality inspection report of salvianolic acid B is available on request to Junlin Cheng.

Sal B was made into freeze-dried powder. The powder was dissolved into 250 mL of 0.9% sodium chloride injection. A placebo was used as a control to eliminate the influence of intravenous infusion and menstruum on subjects. The placebo was 250 mL of 0.9% sodium chloride injection from the market, which was the same batch as the menstruum of the Sal B injection. They were infused intravenously within 60 ± 5 min, and the dripping speed was accurately controlled using the infusion pump.

### 2.2 Study design

This was a single-center, double-blind, randomized placebo-controlled study conducted at the Department of Clinical Pharmacology Lab, Nanjing First Hospital, Nanjing Medical University, Nanjing, China. The study is registered at http://www.chinadrugtrials.org.cn with the identifier CTR20192236, and the study protocol is included therein. According to the animal long-term toxicity test and drug specification of the salvianolic acid B injection, the initial dose is proposed to be 25 mg. From the phase 1 clinical trial of other salvianolic acid salt injection, the maximum tolerance equivalent of salvianolic acid B was 300 mg. The MAD dosage was referred to as the commonly used dose equivalent and the second highest tolerated dose of clinical salvianolic acid salt injection. That was between 150 and 250 mg.

### 2.3 Participants

Healthy Chinese volunteers aged 18–50 years were recruited, whose body mass index was between 19.0 and 26.0 kg/m^2^ and body weight was no less than 50 kg. Key exclusion criteria included any medicine administration in 2 weeks, adoption in another clinical trial during 3 months, donating blood during 3 months prior to the trial, history or clinical evidence of chronic diseases, abnormal results with clinical significance in vital signs, 12-lead electrocardiography, and laboratory examinations of allergic constitution. Volunteers signed the informed consent form before screening.

### 2.4 Randomization and masking

Suitable volunteers enrolled in the study according to the aforementioned criteria. Two volunteers received 25 mg of Sal B, which is the lowest dose in the study. Volunteers were randomized at a ratio of 1:3 in the 75, 200, and 250 mg groups and at a ratio of 1:4 in the 150 and 300 mg groups to receive either a placebo or increasing doses of Sal B in the single-ascending-dose (SAD) study. Other volunteers were randomized at a ratio of 1:3 to receive either a placebo or Sal B in the multiple-ascending-dose (MAD) study. The blind lists were kept under control and would only be uncovered in case of emergency.

### 2.5 Procedures

**FIGURE 1:**
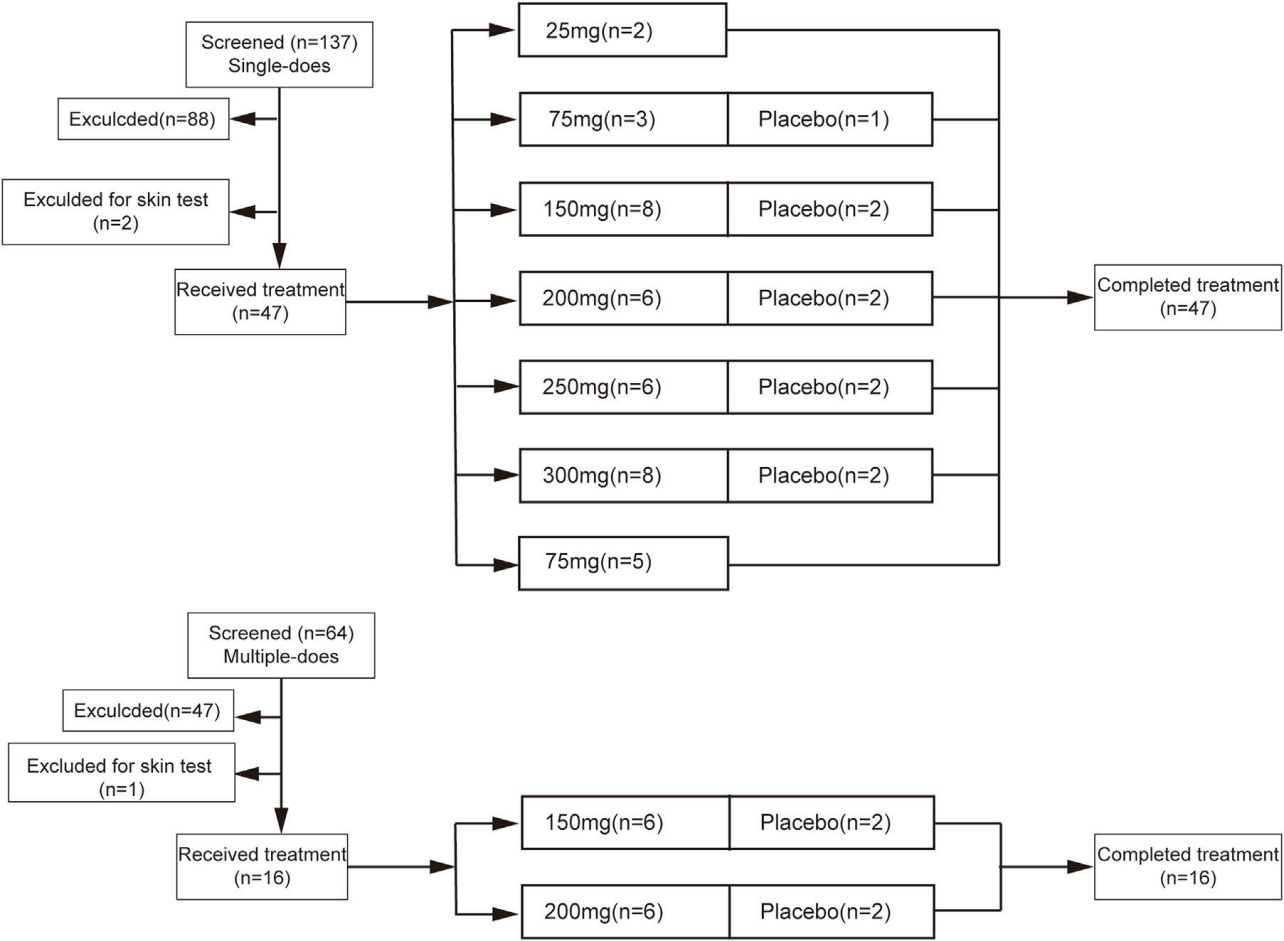
Volunteer disposition.

The study included the SAD study and MAD study (Figure 1). In the SAD study, two eligible volunteers included in the first 25 mg group were treated with Sal B one by one. After the safety was evaluated, the second volunteer was treated with Sal B. Eligible volunteers were randomized to receive the placebo or escalating doses of Sal B (75, 150, 200, 250, and 300 mg) successively. Electrocardiography was taken prior to administration and lasted for 24 h by Holter. The same procedure was conducted in the MAD study. Eligible volunteers were randomized to placebo or Sal B (150, 250 mg) groups for five consecutive days. All the volunteers were hospitalized throughout the trial and came back for a visit on Day 9 ± 1 of the last administration of Sal B. ECG was taken on the 1st and 5th administration days and lasted for 24 h by Holter.

### 2.6 Pharmacokinetics

In the 75, 150, and 300 mg SAD study, blood samples (4 mL each) were taken at 0, 0.25, 0.5, 0.75, and 1 h after the intravenous dose. After the termination of infusion, additional blood samples were taken at 0.17, 0.33, 0.67, 1, 1.5, 2, 3, 4, 5, 6, 7, and 8 h. In the 150 mg MAD study, blood samples were taken at 0, 0.25, 0.5, 0.75, and 1 h after the first dose, 0.17, 0.33, 0.67, 1, 1.5, 2, 3, 4, 5, 6, 7, and 8 h after the termination of the first dose, pre-dose of third and fourth dosing, 0, 0.25, 0.5, 0.75, and 1 h after the last dose, and 0.17, 0.33, 0.67, 1, 1.5, 2, 3, 4, 5, 6, 7, 8, and 24 h after the termination of the last dose. Blood samples were collected in tubes containing ethylenediaminetetraacetic acid dipotassium and centrifuged at 4°C within 1 h after sampling for plasma preparation. Sal B was detected using a liquid chromatography–tandem mass spectrometry method (HPLC 30-AD, SHIMADZU, Japan. AB SCIEX API 4000, Applied Biosystems, United States). The method was validated as per the Chinese Pharmacopoeia.

### 2.7 Safety assessments

Safety and tolerance were determined by clinical evaluation. Volunteers were monitored in hospital from the day before the first administration, and adverse events (AEs) were recorded. All AEs were evaluated in terms of severity (mild, moderate, or severe), duration, measures taken, outcomes, and relationship to treatment. Serious adverse events (SAEs) include death, life-threatening, permanent, or severe disability or functional loss, hospitalization or prolonged hospitalization, and congenital abnormality or birth defect.

### 2.8 Statistical analysis

SAS9.4 software was used for safety analysis. The pharmacokinetic parameters were estimated and analyzed using WinNonlin software (version 8.0) using the non-compartment model. C_max_, T_max_, T_1/2_, AUC_0–24h_, AUC_0-∞_, Vd, and CL/F were calculated in the SAD study, and C_max_, T_max_, AUC_0–24h_, C_av(ss)_, C_max(ss)_, C_min(ss)_, DF, and R_ac_ were calculated in the MAD study.

## 3 Results

### 3.1 Study population

In the SAD study, 47 eligible volunteers were selected from 137 volunteers and assigned to six groups. In the MAD study, 16 eligible volunteers were selected from 64 volunteers and assigned to two groups. All the included volunteers completed the study as planned. The demographics of 63 volunteers are illustrated in Table 1. No significant difference was observed in the age, height, weight, and BMI of all groups.

**TABLE 1:** Volunteer demographics.

Demographic	Sample size	Sex (M/F)	Age (M±SD)	Height (cm, M±SD)	Weight (kg, M±SD)	BMI (kg/m^2^, M±SD)
SAD study						
25 mg	2	2/0	25.00 ± 2.83	176.00 ± 4.24	68.90 ± 5.23	22.30 ± 2.83
75 mg	9	5/4	24.11 ± 2.37	170.67 ± 11.21	64.73 ± 11.41	22.11 ± 2.31
150 mg	10	7/3	29.50 ± 7.41	168.50 ± 8.18	63.64 ± 6.58	22.44 ± 1.96
200 mg	8	4/4	27.00 ± 5.98	165.81 ± 10.23	64.69 ± 12.02	23.31 ± 2.01
250 mg	8	6/2	24.88 ± 2.30	168.94 ± 5.50	65.76 ± 6.03	23.05 ± 1.89
300 mg	10	9/1	24.90 ± 4.28	172.65 ± 8.09	65.50 ± 7.75	21.94 ± 1.86
MAD study						
150 mg	8	6/2	27.13 ± 2.44	171.00 ± 5.53	64.43 ± 6.44	22.05 ± 2.10
250 mg	8	6/2	26.00 ± 5.63	171.31 ± 9.76	67.99 ± 8.06	23.14 ± 1.82

BMI**,** body mass index.

### 3.2 Skin test and allergic reaction

In the study, 66 volunteers received the Sal B injection skin test. The concentration of Sal B in the skin test solution was 1/10 that of Sal B in the corresponding group. There were three volunteers who failed the skin test for enlarged and elevated erythema, and the positive rate was 4.4%. According to the protocol, these three volunteers were excluded from the trial. No skin and systemic allergic reactions were observed in the following procedure in the other 63 volunteers with negative skin tests.

### 3.3 Safety data

In the SAD study, there were 41 AEs in 24 cases with an incidence of 51.1% (24/47). Moreover, 10 treatment-related AEs occurred in nine cases with an incidence of 19.1% (9/47). In the MAD study, there were 13 AEs in eight cases with an incidence of 50.0% (8/16). Furthermore, five treatment-related AEs occurred in four cases with an incidence of 25% (4/16).

Changes in white blood cells occurred in two volunteers who received 75 mg and 250 mg of Sal B. Changes in liver function indicators occurred in 11 volunteers who received 150 mg or more of Sal B. Blood creatinine kinase-MB increased in four volunteers who received 25, 150, 250, and 300 mg of Sal B. Urine N-acetyl-β-D-glucosidase increased in five volunteers, who received 250 mg and 300 mg of Sal B. Blood brain natriuretic peptides increased in four volunteers who received 75, 150, and 300 mg of Sal B. One volunteer who received 200 mg of Sal B felt dizziness, tremor, palpitation, sweat, and low blood pressure but recovered in 40 min. Chest discomfort occurred in one volunteer who received 250 mg of Sal B for 5 consecutive days. Increased triglyceride is the most common adverse event (six cases), followed by increased alanine aminotransferase (five cases), increased NAG (five cases), increased creatinine kinase-MB (four cases), and increased BNP (four cases).

No clinically significant findings regarding physical examination, vital sign, or ECG were observed in volunteers receiving Sal B. No SAEs occurred, and no volunteers withdrew from the trial due to AEs. All observed AEs are shown in Table 2.

**TABLE 2:** Adverse events.

	SAD study						MAD study		Sal B (n = 50)	Placebo (n = 13)
	25 mg (n = 2)	75 mg (n = 8)	150 mg (n = 8)	200 mg (n = 6)	250 mg (n = 6)	300 mg (n = 8)	150 mg (n = 6)	250 mg (n = 6)		
Adverse events of any cause, n (%)	1 (50.0)	6 (75.0)	2 (25.0)	2 (33.3)	3 (50.0)	6 (75.0)	2 (33.3)	4 (66.7)	26 (52.0)	5 (38.5)
Laboratory examination										
White blood cell counts decreased	0	2 (25.0)	0	0	0	0	0	0	2 (4.0)	0
White blood cell counts increased	0	0	0	0	2 (33.3)	0	0	0	2 (4.0)	0
Blood alanine aminotransferase increased	0	0	0	1 (16.7)	0	1 (12.5)	1 (16.7)	2 (33.3)	5 (10.0)	0
Blood glutamyl transpeptidase increased	0	0	0	1 (16.7)	0	0	0	0	1 (2.0)	0
Blood aspartate aminotransferase increased	0	0	0	1 (16.7)	0	1 (12.5)	0	1 (16.7)	3 (6.0)	1 (7.7)
Blood alkaline phosphatases increased	0	0	0	1 (16.7)	0	0	0	0	1 (2.0)	0
Blood bilirubin increased	0	0	1 (12.5)	0	0	0	0	0	1 (2.0)	0
Blood triglycerides increased	0	0	0	1 (16.7)	0	2 (25.0)	1 (16.7)	2 (33.3)	6 (12.0)	0
Blood creatinine kinase-MB increased	1 (50.0)	0	1 (12.5)	0	1 (16.7)	1 (12.5)	0	0	4 (8.0)	1 (7.7)
Blood D-dimer increased	0	1 (12.5)	0	0	0	0	0	0	1 (2.0)	1 (7.7)
Urine N-acetyl-β-D-glucosidase increased creased increased	0	0	0	0	1 (16.7)	2 (25.0)	0	2 (33.3)	5 (10.0)	2 (15.4)
Blood fasting blood glucose increased	0	0	0	0	0	1 (12.5)	0	0	1 (2.0)	0
Blood brain natriuretic peptides increased	0	1 (12.5)	1 (12.5)	0	0	2 (25.0)	0	0	4 (8.0)	0
Assistant examination										
Ventricular extrasystoles	0	0	0	0	0	0	0	0	0	1 (7.7)
Symptoms and signs										
Dizziness	0	0	0	2 (33.3)	0	0	0	0	2 (4.0)	1 (7.7)
Tremor	0	0	0	1 (16.7)	0	0	0	0	1 (2.0)	0
Palpitation	0	0	0	1 (16.7)	0	0	0	0	1 (2.0)	0
Sweat	0	0	0	1 (16.7)	0	0	0	0	1 (2.0)	0
Low blood pressure	0	0	0	1 (16.7)	0	0	0	0	1 (2.0)	0
High blood pressure	0	0	0	0	0	1 (12.5)	0	0	1 (2.0)	0
Upper respiratory tract infection	0	2 (25.0)	0	0	0	0	1 (16.7)	0	3 (6.0)	0
Chest discomfort	0	0	0	0	0	0	0	1 (16.7)	1 (2.0)	0
Treatment-related adverse events, n (%)	0	1 (12.5)	2 (25.0)	1 (16.7)	0	2 (25.0)	1 (16.7)	3 (50.0)	10 (20.0)	3 (23.1)
Laboratory examination										
Blood alanine aminotransferase increased	0	0	0	0	0	0	1 (16.7)	1 (16.7)	2 (4.0)	0
Blood bilirubin increased	0	0	1 (12.5)	0	0	0	0	0	1 (2.0)	0
Blood creatinine kinase-MB increased	0	0	1 (12.5)	0	0	0	0	0	1 (2.0)	0
Blood brain natriuretic peptides increased	0	1 (12.5)	1 (12.5)	0	0	2 (25.0)	0	0	4 (8.0)	0
Urine N-acetyl-β-D-glucosidase increased	0	0	0	0	0	0	0	2 (33.3)	2 (4.0)	1 (7.7)
Assistant examination										
Ventricular extrasystoles	0	0	0	0	0	0	0	0	0	1 (7.7)
Symptoms and signs										
Dizziness	0	0	0	1 (16.7)	0	0	0	0	1 (2.0)	1 (7.7)
Chest discomfort	0	0	0	0	0	0	0	1 (16.7)	1 (2.0)	0

Data are represented as n (%). “Treatment-related adverse events” is defined as the relationship of an adverse event to the study drug being potential, probable, or definitive.

### 3.4 Pharmacokinetics

**FIGURE 2:**
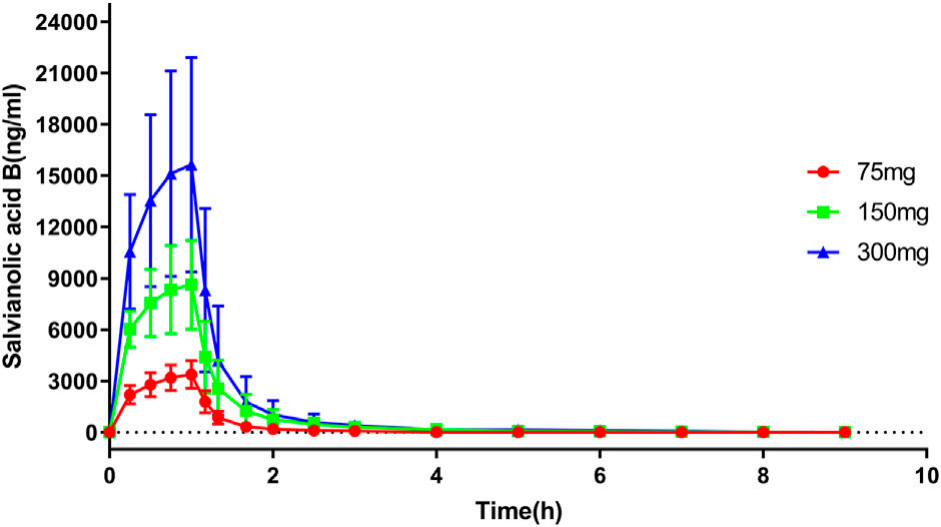
Plasma concentration–time profile of salvianolic B in the SAD study. *n* = 8. Data are presented as the mean ± SD.

After a single administration of Sal B injection, the plasma exposure of Sal B increased in proportion to the dose, approximately. The mean C_max_ was 3431, 8646, and 15,925 ng/mL, and the mean AUC_0-t_ was 3576, 10,237, and 17,841 h*ng/mL in the 75, 150, and 300 mg groups. The plasma concentration–time profiles of Sal B are shown in Figure 2.

**FIGURE 3:**
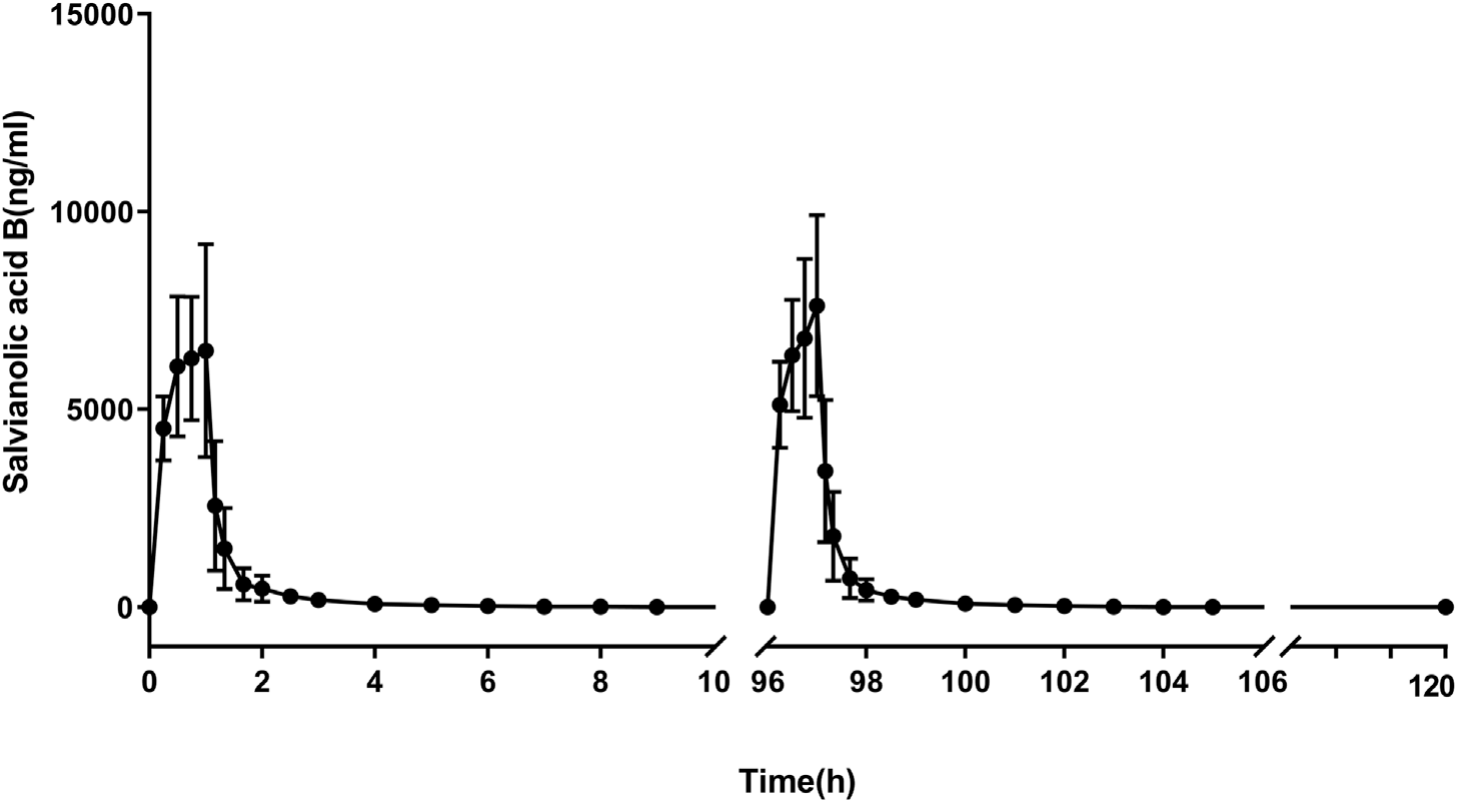
Plasma concentration–time profile of salvianolic B in the MAD study. *n* = 6. Data are presented as the mean ± SD.

After 5-consecutive-day administration of Sal B, C_av(ss)_ ranged from 227 ng/mL to 348 ng/mL, and C_max(ss)_ ranged from 5010 to 10,700 ng/mL. The mean C_max_ was 6875 and 7621 ng/mL, and the mean AUC_0-t_ was 7365 and 8227 h*ng/mL in the first and last doses of Sal B. The accumulation coefficient (R_ac_) is 1.0. The plasma concentration–time profiles and pharmacokinetic parameter data are shown in Figure 3 and Table 3.

**TABLE 3:** Pharmacokinetic parameters.

	SAD study			MAD study	
	75 (n = 8)	150 (n = 8)	300 (n = 8)	First dose (n = 6)	Last dose (n = 6)
T_max_, h	0.95 ± 0.12	0.97 ± 0.09	0.95 ± 0.12	0.96 ± 0.22	1.03 ± 0.01
T_1/2_, h	0.67 ± 0.27	1.45 ± 0.50	2.13 ± 0.73	-	-
Cmax, ng/mL	3431 ± 781	8646 ± 2580	15,925 ± 6082	6875 ± 1937	7621 ± 2288
AUC_0-t_, h*ng/mL	3576 ± 966	10,237 ± 3992	17,841 ± 8071	7365 ± 2722	8227 ± 2854
AUC_0-∞_, h*ng/mL	3660 ± 995	10,404 ± 4025	17,095 ± 8113		
CL, mL/h	22,477 ± 8810	16,266 ± 5630	18,876 ± 6474		
Cav_(ss)_, ng/mL					348 ± 121
DF, %				-	2218 ± 137
Rac				-	1.0

T_max_, time to peak plasma concentration; T_1/2_, terminal elimination half-life; C_max_, peak plasma concentration; AUC_0-t_, area under the plasma concentration–time curve from time zero to time t; AUC_0-∞_, area under the plasma concentration–time curve from time zero to infinity; CL, clearance; DF, dissipation factor; Rac, accumulation ratio; Cav(ss), average steady state concentration.

## 4 Discussion

The purpose of this phase 1 clinical trial was to evaluate the safety, tolerability, and pharmacokinetics of Sal B injection in healthy volunteers. Overall, Sal B injection was well tolerated in healthy Chinese volunteers (25–300 mg), and the pharmacokinetic data exhibited dose-proportional increase in the 75, 150, and 300 mg groups. The MAD study revealed no accumulation. All the AEs were mild and resolved without any medical treatment, and continuous Sal B injection administration was possible. The physiological conditions of the volunteers were stable throughout the trial.

TCM injections in clinical application were usually the extracted mixture of TCMs, which were prone to cause allergic reaction because of their low purity (Xu and Dou, 2015). On the market, about 80% of the extracted mixture of salvianolic acid injection is salvianolic acid, while in this study the extract contains Sal B up to 96%. Only three cases of erythema were observed in the skin test, and no allergic reaction was observed in the following trial, which is probably due to the small amount of impurities.

Sal B is methylated by the liver to produce active metabolites, which may cause potential hepatotoxicity. Therefore, serum biochemical indicators should be concerned (Jia et al., 2010; Cheng et al., 2020). Blood alanine aminotransferase, glutamyl transpeptidase, aspartate aminotransferase, alkaline phosphatase, and/or bilirubin increased in 11 volunteers treated with Sal B, while only aspartate aminotransferase increased in the placebo group. The indicators increased within twice of the upper limit and recovered to the baseline in several days, indicating slight damage to the liver. A real-world study in SM depside salt revealed the same mild liver dysfunction in patients (Yan et al., 2017).

No significant damage of Sal B on renal function was found (Du et al., 2020). As a sensitive indicator of acute tubular injury, NAG increased in five volunteers with Sal B administration and once in the placebo group, which recovered in less than 10 days. Therefore, the damage of Sal B on the kidney seems to be slight and transient.

The elevated BNP is a marker of heart failure, and it is also a protective hormone to decrease blood pressure. We found the elevated BNP in four volunteers treated by Sal B, which was consistent with preclinical studies in healthy rats (Meng et al., 2018). Several TCMs showed dual-directional regulation *in vivo*, so we believe that Sal B has the same mechanism in healthy volunteers (Huo et al., 2000). In this study, the recipients enrolled were healthy volunteers with an average age of 26.2 years; further studies of the Sal B injection should be conducted in patients with cardiovascular disease.

Dizziness was the most common symptom among AEs (two cases, 4.0%) in Sal B groups, which is consistent with other reports (Ling et al., 2012; Yan et al., 2017). Two cases were mild and occurred at about 2 and 7 h after dosing and were experienced for 3 and 1.5 h, respectively, in the 200 mg group. Tremor, palpitation, sweat, and low blood pressure (SBP88mmHg/DBP49 mmHg) occurred in one volunteer during the same period, which were possibly complicated symptoms caused by dizziness. Chest discomfort (one cases, 2.0%) and increased creatinine kinase-MB (four cases, 8.0%) may be mediated by cardiovascular system damage, which were also found in other studies (Cong et al., 2022). Consistent with other studies (Ling et al., 2012), no clinical abnormalities were recorded in 12-lead ECG and Holter. All the clinical symptoms and signs were mild. Sal B showed a little effect on the coagulation system (Yan et al., 2017; Cong et al., 2022).

C_max_ and AUC of Sal B proportionally increased with the dose in the 75, 150, and 300 SAD study. C_max_ of the first (d1) and last doses (d5) in the 150-mg MAD study and the 150-mg SAD group were basically the same (*p* = 0.56, 0.46, and 0.19). The AUC_0–t_ showed no significant difference between the first (d1) and last doses (d5) in the MAD phase and the 150-mg SAD group (*p* = 0.55, 0.31, and 0.16). The accumulation index was 1.0, which revealed no Sal B accumulated *in vivo*.

In conclusion, the current study showed that SAD at 25–300 mg and MAD up to 250 mg of Sal B were well tolerated with no SAEs observed. The accumulated safety and PK data in healthy volunteers support further evaluation in patient’s clinical trials for the treatment of Sal B.

## Data Availability

The original contributions presented in the study are included in the article/Supplementary Material; further inquiries can be directed to the corresponding authors.
